# Supplementary material for: Nutritional Value and Contaminant Risk Assessment of Some Commercially Important Fishes and Crawfish of Lake Trasimeno, Italy
Source: Int J Environ Res Public Health. 2020 Apr 8;17(7):2545. doi: 10.3390/ijerph17072545 (PMC7177714; doi:10.3390/ijerph17072545)
Supplement: Supplementary file 1 [file ijerph-17-02545-s001.pdf]

International Journal of
Environmental Research and Public Health

MDPI

Table S1. Comprehensive list of pesticides, metals, and PCB analyzed. The number of tested samples and the number of positive samples (> LOQ) are reported.

	Tested samples	Positive samples		Tested samples	Positive samples
**Pesticides**					
Acrinatrin	34		Oxychlordane	34	
Aldrin	39		Paration	39	
Azinphos-ethyl	20		Paration-methyl	39	
Bifentrin	20		Pendimethalin	34	5
Ciflutrin	39		Procymidone	5	
Cipermetrin	39		Profenofos	39	
Cis-clordano	39		Quintozen	34	
ɣ-Chlordane	39		Tecnazen	34	
Chlorobenzilate	15		Triazofos	39	
Chlorpyriphos	34		Vinclozolin	34	
Deltametrin	39				
Diazinon	39		**Metals**		
Dicofol	39		Pb	69	18
Dieldrin	39		Cd	67	20
2,4-DDD	39		Hg	68	65
2,4-DDE	39		Ni	28	17
2,4-DDT	39		Cr	29	3
4,4-DDD	39		As	30	26
4,4-DDE	39	3			
4,4-DDT	39		**PCB**		
Heptachlor endo ep.	39		PCB 28	31	26
α- HCH	39		PCB 52	31	30
β- HCH	39		PCB 101	31	31
ɣ- HCH	39		PCB 153	31	31
Etofenprox	15		PCB 138	31	31
Fenopropatrin	39		PCB 180	31	31
Fention	39				
Fenvalerate	39				
Flucitrinate	39				
HCB	39				
ƛ-Cyhalothrin	39				
Malation	39				
Metacrifos	39				
Metidation	39				
Methoxychlor	39				
Nitrofen	15				
Oxychlordane	34				
Paration	39				

CC BY

© 2020 by the authors. Licensee MDPI, Basel, Switzerland. This article is an open access article distributed under the terms and conditions of the Creative Commons Attribution (CC BY) license (http://creativecommons.org/licenses/by/4.0/).
